# The value of multimodal ultrasound imaging in differentiating HER2-positive breast cancer

**DOI:** 10.3389/fonc.2026.1718785

**Published:** 2026-01-29

**Authors:** Yu-Meng Lei, Chen Liu, Bing-Song Lei, Chen Liao, Ning Zhang, Qi Wang, Shu-E Zeng, Ge Zhang, Hua-Rong Ye

**Affiliations:** 1 Department of Medical Ultrasound, China Resources and Wisco General Hospital, Wuhan University of Science and Technology, Wuhan, China; 2 Medical College, Wuhan University of Science and Technology, Wuhan, China; 3 Department of Medical Ultrasound, Hubei Cancer Hospital, Tongji Medical College, Huazhong University of Science and Technology, Wuhan, China; 4 Breast Cancer Center, Hubei Cancer Hospital, National Key Clinical Specialty Discipline Construction Program, Hubei Provincial Clinical Research Center for Breast Cancer, Wuhan, China

**Keywords:** breast cancer, contrast-enhanced ultrasound, differential diagnosis, HER2-positive, super-resolution ultrasound

## Abstract

**Objectives:**

This study investigated vascular differences between human epidermal growth factor receptor 2 (HER2)-positive and HER2-negative breast cancers using multimodal ultrasound and evaluated their diagnostic utility.

**Methods:**

Breast masses were examined with conventional ultrasound (CUS), color Doppler flow imaging (CDFI), contrast-enhanced ultrasound (CEUS), and super-resolution ultrasound (SRUS). Quantitative and qualitative parameters were extracted. Binary logistic regression was used to identify independent predictors of HER2-positive breast cancer. The diagnostic performance was evaluated by receiver operating characteristic (ROC) curve.

**Results:**

72 breast masses were divided into HER2-positive (n = 23) and HER2-negative (n = 49) groups according to immunohistochemical results. Univariate analysis showed that 12 parameters of four ultrasound modalities were significantly different between the groups (p < 0.05), whereas multivariate analysis showed differences in 8 parameters. Maximum diameter of the lump, microcalcification, peak intensity and max flow velocity of HER2-positive group were significantly greater than HER2-negative group (*p* < 0.05). HER2-positive group showed greater lesion extent after CEUS, less mean tortuosity, more uniform microvasculature distribution and microvasculature flow direction was away from the transducer (*p* < 0.05). The combination of CUS, CEUS, and SRUS achieved the highest area under the curve (AUC) of 0.976, with sensitivity, specificity and accuracy of 95.7%, 93.9% and 94.4%, respectively.

**Conclusions:**

Multimodal ultrasound parameters from CUS, CEUS, and SRUS can predict HER2-positive breast cancer, and their combination provides superior diagnostic efficacy.

## 1 Introduction

Breast cancer is the leading cause of cancer death among women worldwide, with approximately 2.3 million new cases in 2022, accounting for 25% of all female cancers (1). Different molecular subtypes of breast cancer vary in malignancy and treatment, making early identification of molecular subtypes critical for patient management (2). The human epidermal growth factor receptor 2 (HER2) is a tyrosine kinase receptor (3). When the HER2 gene is amplified and its related kinase receptor protein is overexpressed, the cell proliferation regulation process is dysregulated, thereby promoting cancer cell proliferation and invasion, and then leading to the occurrence of malignant tumors. Compared to HER2-negative breast cancer, HER2-positive breast cancer has the characteristics of fast growth, strong invasiveness, easy visceral metastasis, and poor prognosis, accounting for 25% of all breast cancer cases (4, 5). Therefore, early identification and treatment of HER2-positive breast cancer is essential to improve patient prognosis.

Angiogenesis is a critical hallmark of breast cancer invasiveness and plays a central role in tumor growth and metastasis (6). Studies have shown that the generation of tumor angiogenesis has been considered an early sign of cancer, and it is closely related to the expression of vascular endothelial growth factor (VEGF) (7). Previous studies have shown that HER2 can upregulate VEGF, promoting neovascularization within tumor tissues (8). Related research results show that there are marked differences in neovascular among breast cancers with different HER2 expression levels (9). Therefore, early noninvasive evaluation of tumor microvasculature has significant clinical value for HER2 detection and prognosis assessment.

Common imaging techniques, including magnetic resonance imaging (MRI) and contrast-enhanced ultrasound (CEUS), provide information on breast tumor vascularity. MRI offers high sensitivity but limited spatial resolution, restricting microvasculature assessment (10, 11). Conventional ultrasound (CUS), with advantages of real-time imaging, radiation-free operation, accurate localization, and simplicity, is widely used for breast screening in Chinese women (12). CUS provides morphological information on breast masses, including size, shape, margin, and internal echotexture. Color Doppler flow imaging (CDFI) evaluates tissue perfusion, visualizing large vessels and rapid flow within and around the mass (13–16). CEUS uses microbubble contrast agents to real-time dynamic display of tumor blood perfusion, including small, low-flow vessels (17–19). Super-resolution ultrasound (SRUS) breaks through the acoustic diffraction limit, enabling visualization of microvasculature with spatial resolutions on the order of tens of micrometers. This technique enables noninvasive visualization of the tumor microvasculature network and provides detailed information on both its morphology and hemodynamic characteristics (20–22). Meanwhile, SRUS has been validated in preclinical tumour models and clinical pilot studies (23), and our research group has previously confirmed that it can clearly display the microvasculature system in human breast and thyroid lesions (24, 25). However, further research is needed in areas such as molecular typing of breast cancer.

In this study, we used CUS, CDFI, CEUS, and SRUS to extract morphological and vascular features of breast masses. We assessed the correlation between multimodal ultrasound parameters and HER2-positive breast cancer and developed a predictive model to aid individualized treatment and improve breast cancer management.

## 2 Method

### 2.1 Patient population

**Figure 1:**
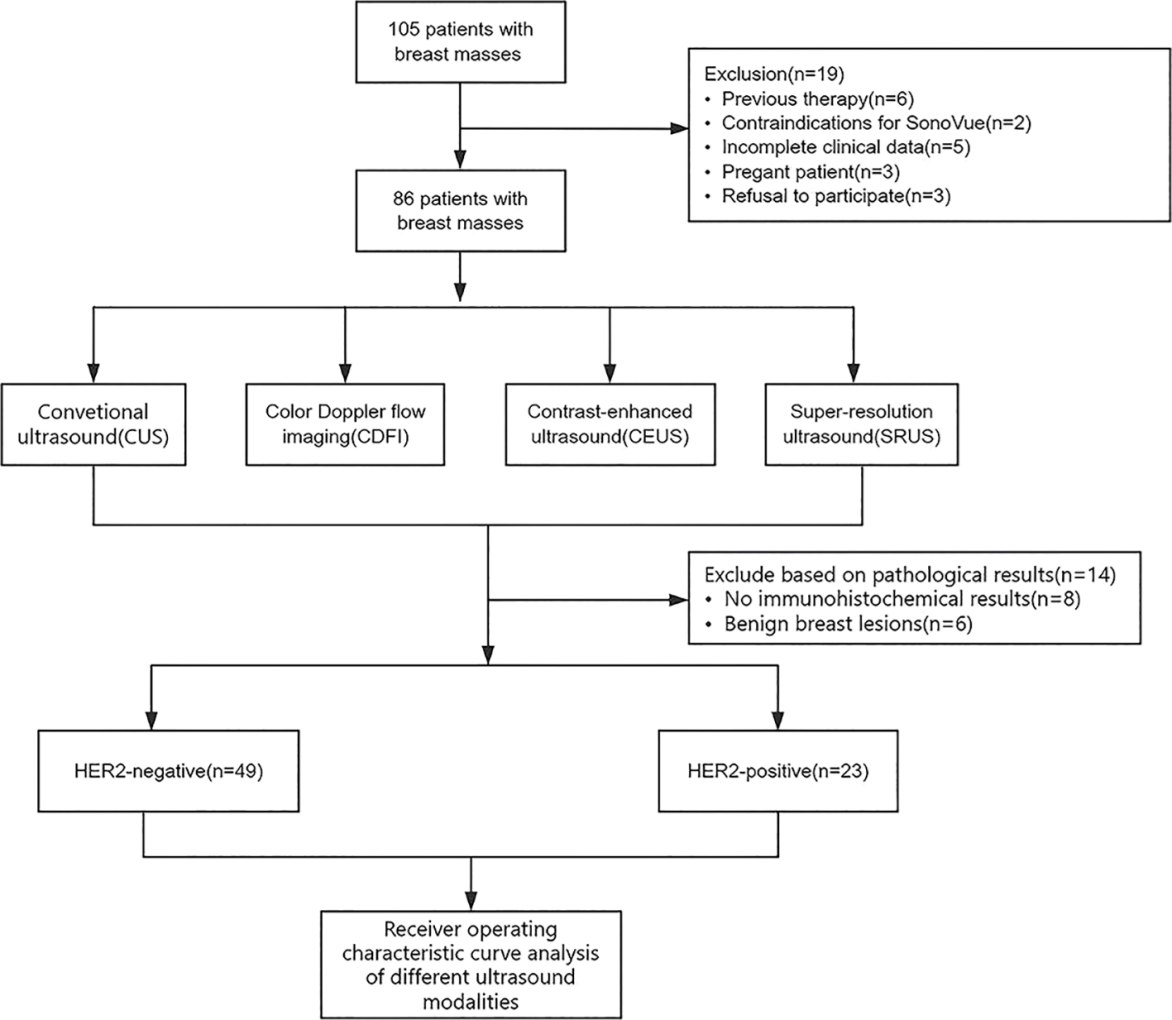
Patient enrollment and data processing pipeline.

We conducted a prospective, dual-center study including 105 patients with suspected malignant breast tumors from China Resources & Wisco General Hospital and Hubei Cancer Hospital between January and December 2023. All patients underwent multimodal ultrasound examinations, including CUS, CDFI, and CEUS. Inclusion criteria: (1) surgical resection with pathologically confirmed malignancy; (2) comprehensive immunohistochemistry (IHC) results clearly distinguishing HER2-positive and HER2-negative cases; (3) surgery performed within one month after ultrasound; (4) no prior neoadjuvant chemotherapy or other treatments before ultrasonography. Exclusion criteria: (1) previous therapy; (2) contraindications for SonoVue; (3) incomplete clinical data; (4) pregnancy; (5) refusal to participate. The required sample size was calculated *a priori* using PASS 2025 Efficacy Analysis and Sample Size Software (Version 2025). A two-tailed, two-sample t-test (unequal variances) was employed with a significance level of 0.05 and a test power of 0.80. Based on the anticipated effect size, a minimum of 50 patients is required. Considering a potential 20% dropout rate, at least 63 patients should be enrolled. IHC results of post-surgical breast masses served as the gold standard. This study was approved by the Medical Ethics Committee of hospitals, and all participants provided written informed consent. **Figure 1** illustrates patient registration and data processing.

### 2.2 HER2-positive breast cancer diagnosis

HER2 expression was assessed using the EnVision™ IHC method and classified as four different levels (-, +, ++, and +++). HER2 (-) and HER2 (+) are classified as HER2-negative, while HER2 (+++) is classified as HER2-positive. HER2 (++) is considered a borderline result. In this case, fluorescence *in situ* hybridization (FISH) is performed to check for amplification of HER2. If HER2 amplification is not found, HER2 (++) is classified as HER2-negative. If HER2 amplification occurs, HER2 (++) is classified as HER2-positive. In this study, three cases initially showed IHC 2+ results. Further FISH testing revealed no HER2 amplification in any of these cases, leading to their reclassification as HER2-negative.

### 2.3 Ultrasonic data acquisition

All ultrasounds were performed by physicians with over 20 years of experience using Mindray Resona R9 with an L11-3U linear probe (3–10 MHz). Patients were supine with arms raised. CUS was performed to assess the gray-scale characteristics and maximum diameter of the lesion. CDFI was used to evaluate vascularity, and the plane showing the richest blood flow, including both the lesion and surrounding tissue, was selected. CEUS was performed using a mechanical index of 0.08. Subsequently, 0.5 mL of SonoVue (Bracco, Milan, Italy) microbubble contrast agent was injected via the cubital peripheral vein, immediately followed by a 5 mL saline flush. More than 1000 frames of ultrasound contrast images were collected at a frame rate of approximately 80 Hz for further processing and analysis by ultrasound super-resolution imaging. Real-time dual-mode images (CUS and CEUS) were used to guide the imaging plane and monitor the microbubble signal after injection. The imaging plane remained unchanged to ensure that all images were acquired in the same area. The remaining 4.3 mL of SonoVue microbubble contrast agent was injected through the cubital peripheral vein and immediately flushed with 5 mL of normal saline. After the injection, the timer and dynamic storage function of the ultrasound instrument were turned on to collect conventional ultrasound contrast images for subsequent analysis.

### 2.4 Ultrasonic multimodal parameter extraction

Maximum diameter of the lump, boundary, morphology, internal echo, orientation, microcalcification, and posterior echo attenuation were obtained from greyscale images. CDFI images were independently graded by a radiologist using the Adler grading system (grade 0: no detectable flow; grade 1: 1–2 punctate signals; grade 2: 3–4 punctate signals or a short vessel-like flow; grade 3: ≥5 punctate signals or ≥2 vessel-like flows). CEUS data were analyzed using the system software, with regions of interest (ROIs) placed in the most enhanced lesion area and corresponding normal tissue to generate Time–intensity curves (TICs) parameters including time to peak, peak intensity (PI), wash-in rate, wash-out rate and area under the curve (AUC). Each CEUS quantitative parameter was measured three times using the system software, and the average value was used for subsequent analysis. Qualitative CEUS features were assessed based on standardized visual evaluation and include: enhancement time (rapid, simultaneous, or delayed), enhancement intensity (hypoenhancement, isoenhancement, or hyperenhancement), enhancement pattern (centripetal, centrifugal, or diffuse), contrast agent distribution (homogeneous or heterogeneous), enhancement margin (clear or blurred), filling defects (presence or absence of non-enhanced areas), peripheral vessels penetrating the lesion (present or absent), and area of enhancement (unchanged or larger). High-frame-rate CEUS data were exported from the ultrasound device in DICOM format and processed offline using MATLAB (MathWorks Inc., Natick, MA, USA) for super-resolution ultrasound (SRUS) analysisreconstruction (26). SRUS required singular value decomposition (SVD) to separate tissue and background signals from microbubble (MB) signals. Following SVD filtering, a pixel intensity threshold is applied to isolate MB signals, and the area, intensity, and shape/eccentricity of each MB are measured to eliminate artifacts. The spatial coordinates of individual MBs are determined using an intensity-weighted centroid method, which locates each MB signal by calculating the center of its intensity distribution. Localization results from all frames are aggregated to reconstruct a super-resolution microvasculature map. Microvasculature density (MVD) is calculated as the total tracked MB area divided by the ROI. The ROI is manually delineated in MATLAB to align with lesions visible on CUS images and corresponding super-resolution velocity maps. Microvasculature flow velocity is obtained through continuous inter-frame MB tracking. For each MB in frame F, the best-matched MB in frame F + 1 is identified within a 700 μm search window. At an 80 Hz frame rate, velocities up to 40 mm/s can be tracked. MB pairs are accepted if the maximum normalized cross-correlation between frames exceeds a threshold of 0.9; pairs outside the search window or failing the threshold are excluded. Using this method, multiple quantitative parameters—including mean and max flow velocity, mean and max tortuosity, mean and largest diameter, fractal dimension—are calculated by tracking microbubbles (MBs). Results within ROIs are averaged to comprehensively characterize the tumor microvasculature system. In addition to quantitative analysis, qualitative SRUS parameters, including microvasculature distribution and microvasculature flow direction, were assessed based on the reconstructed super-resolution microvasculature maps. The Adler grade and all qualitative parameters were assessed independently by two doctors. In case of disagreement, a third doctor was consulted for assessment. Detailed definitions and calculation methods of CEUS and SRUS parameters are summarized in **Table 1**, while the interobserver agreement results are presented in **Table 2**.

**Table 1:** Definitions and measurement methods of CEUS and SRUS parameters.

Parameter	Definition and measurement	Calculation formula
PI	The maximum intensity of the enhancing curve during the bolus	(postcontrast signal-precontrast signal)/precontrast signal
TTP	The time from the appearance of the first microbubbles in the lesion to its maximum peak intensity	t(peak intensity)−t(contrast arrival)
AUC	Total area under the time–intensity curve	NA
Wash-in rate	Slope of the ascending portion of the curve during contrast wash-in	NA
Wash-out rate	Slope of the descending portion of the curve during contrast wash-out	NA
Wash-in rate/wash-out rate	Describe the relative velocity of contrast agent inflow and outflow	NA
Enhancement time	Time interval from contrast injection to initial appearance of microbubbles in the lesion	NA
Enhancement intensity	Degree of contrast signal enhancement within the lesion	NA
Enhancement pattern	Spatial and temporal distribution characteristics of contrast-enhanced lesions	NA
Distribution of contrast agent	Distribution of microbubbles within the lesion	NA
Enhancement margin	The morphology of the lesion margins during enhancement	NA
Filling defect	Areas within the lesion lacking contrast uptake, indicating possible necrosis or avascular regions	NA
Peripheral vessels penetrating the lesion	Peripheral vessels entering the interior from around the lesion	NA
Area of enhancement	Comparison of the maximum diameter of the lump on the contrast-enhanced ultrasound image with that on the corresponding conventional ultrasound image	NA
Microvasculature flow velocity	The average speed of microbubble movement within the reconstructed microvasculature network	NA
Microvasculature tortuosity	The degree of curvature and twisting of microvessels	NA
Microvasculature diameter	The local or average width of microvessels extracted from images	NA
Fractal dimension	The geometric complexity and self-similarity of the microvasculature network	NA
MVD	Tracked microbubble area divided by the ROI area of mass	tracked microbubble area divided by the ROI area of mass
Microvasculature distribution	The spatial arrangement and density distribution of microvessels within the lesion.	NA
Microvasculature flow direction	The primary direction of blood flow in the microvasculature network	NA

**Table 2:** The measures of interobserver agreement between the two observers.

Parameter	Interobserver agreement (%)	Kappa value	95% C.I.	*P*
Boundary	92	0.820	0.701-0.899	<0.001
Morphology	90	0.792	0.671-0.879	0.003
Internal echo	85	0.772	0.651-0.860	0.062
Orientation	90	0.854	0.742-0.920	<0.001
Microcalcification	88	0.761	0.639-0.849	0.012
Posterior echo attenuation	89	0.789	0.667-0.877	0.004
Adler grade	90	0.805	0.685-0.891	0.001
Enhancement time	85	0.749	0.624-0.835	0.134
Enhancement intensity	88	0.762	0.641-0.850	0.018
Enhancement pattern	86	0.735	0.611-0.823	0.034
Distribution of contrast agent	88	0.760	0.638-0.848	0.020
Enhancement margin	83	0.771	0.650-0.859	0.152
Filling defect	87	0.742	0.617-0.830	0.042
Peripheral vessels penetrating the lesion	88	0.754	0.632-0.842	0.021
Area of enhancement	89	0.770	0.649-0.858	0.009
Microvasculature distribution	90	0.788	0.666-0.876	0.005
Microvasculature flow direction	91	0.801	0.680-0.888	0.002

### 2.5 Statistical analysis

SPSS 27.0 software was used for statistical analysis of all data. Interobserver agreement for qualitative parameters was assessed using Cohen’s kappa statistics. Continuous variables were tested for normality and homogeneity of variance. Two-sample t-tests were applied for normally distributed data with equal variance; Mann–Whitney U tests for non-normal or unequal variance data. Categorical variables were compared using chi-square or Fisher’s exact tests. Logistic regression was used to select independent variables, and significant parameters were incorporated to build a HER2-positive prediction model. IHC results served as the gold standard to calculate sensitivity and specificity, and the receiver operating characteristic curve was drawn. The receiver operating characteristic (ROC) curve and AUC were used to evaluate diagnostic performance of CUS, CDFI, CEUS, SRUS, and their combinations. Statistical significance was defined as p < 0.05, with significance levels noted at 0.05 (*) and 0.01 (**) (two-tailed).

## 3 Result

### 3.1 Ultrasound images of breast masses

**Figure 2:**
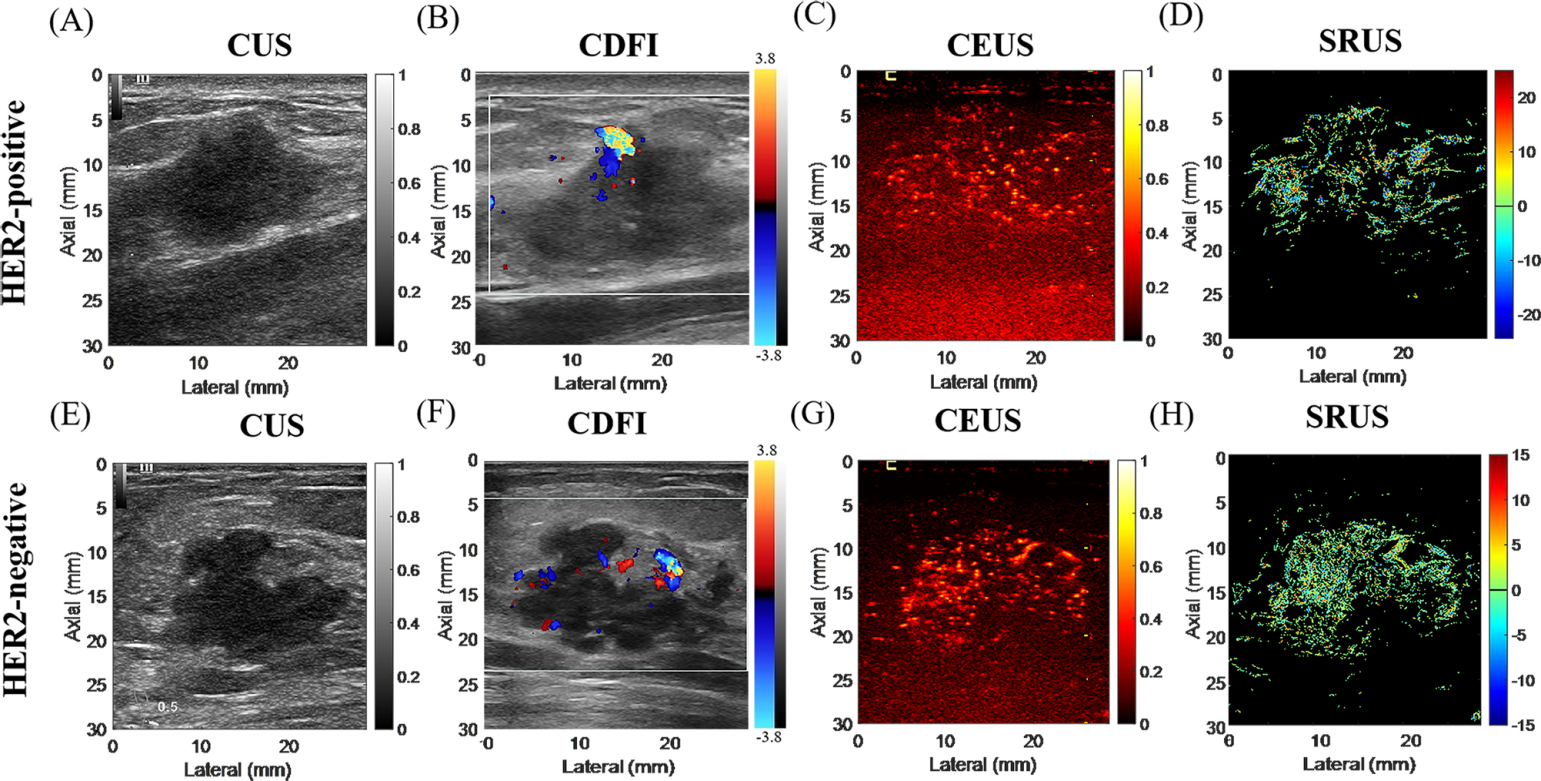
Representative multimodal ultrasound images of breast masses with HER2-positive and HER2- negative. **(A–D)** correspond to a HER2-positive case: **(A)** conventional ultrasound image; **(B)** color Doppler flow image; **(C)** contrast-enhanced ultrasound image; **(D)** super-resolution ultrasound image. **(E–H)** correspond to a HER2-negative case: **(E)** conventional ultrasound image; **(F)** color Doppler flow image; **(G)** contrast-enhanced ultrasound image; **(H)** super-resolution ultrasound image.

We ultimately included 72 patients, comprising 23 HER2-positive patients (mean age, 52.04 ± 8.93 years) and 49 HER2-negative patients (mean age, 53.55 ± 9.16 years). **Figure 2** showed CUS, CDFI, CEUS, and SRUS images of a patient with HER2-positive and HER2-negative breast masses, respectively.

### 3.2 Comparison of clinicopathological characteristics, CUS and CDFI parameters between HER2-positive and HER2-negative breast cancers

Significant differences between HER2-positive and HER2-negative breast cancers were observed in maximum diameter of the lump, orientation, and microcalcification. HER2-positive breast cancers exhibited a significantly larger maximum diameter compared to HER2-negative tumors (3.78 ± 1.38 cm vs. 3.07 ± 1.34 cm, *p* = 0.043). In terms of orientation, a higher proportion of HER2-positive breast cancer showed a parallel growth pattern relative to the skin (87.0% vs. 59.2%, *p* = 0.018). Additionally, microcalcification was more frequently detected in the HER2-positive group (65.2% vs. 32.7%, *p* = 0.009). No significant associations were found for patient age, histologic grade, lymph node status, boundary, morphology, internal echo, posterior echo attenuation, or Adler grade. The results are shown in **Table 3**.

**Table 3:** Clinicopathological characteristics and CUS and CDFI ultrasound parameters in HER2-positive and HER2-negative breast cancers.

Parameters	HER2-positive (n=23)	HER2-negative (n=49)	t/χ²	*P*
Patient age	52.04 ± 8.93	53.55 ± 9.16	0.656	0.514
Maximum diameter of the lump (cm)	3.78 ± 1.38	3.07 ± 1.34	-2.058	**0.043**
Histologic grade			2.510	0.113
Grade I and II	11 (47.8)	33 (67.3)		
Grade III	12 (52.2)	16 (32.7)		
Lymph node status			0.888	0.346
Negative	14 (60.9)	24 (49.0)		
Positive	9 (39.1)	25 (51.0)		
Boundary			1.226	0.268
Clear	8 (34.8)	11 (22.4)		
Blurring	15 (65.2)	38 (77.6)		
Morphology			0.566	0.452
Regular	6 (26.1)	9 (18.4)		
Irregular	17 (73.9)	40 (81.6)		
Internal echo			0.006	0.939
Non-hypoechoic	2 (8.7)	4 (8.2)		
Hypoechoic	23 (91.3)	45 (91.8)		
Orientation			5.554	**0.018**
Parallel	20 (87.0)	29 (59.2)		
Vertical	3 (13.0)	20 (40.8)		
Microcalcification			6.770	**0.009**
No	8 (34.8)	33 (67.3)		
Yes	15 (65.2)	16 (32.7)		
Posterior echo attenuation			0.026	0.872
No	15 (65.2)	31 (63.3)		
Yes	8 (34.8)	18 (36.7)		
Adler grade			0.541	0.910
0	1 (4.3)	1 (2.0)		
1	4 (17.4)	9 (18.4)		
2	10 (43.5)	19 (38.8)		
3	8 (34.8)	20 (40.8)		

Bold values indicate statistically significant differences (P < 0.05).

### 3.3 Comparison of CEUS and SRUS parameters between HER2-positive and HER2-negative breast cancers

**Figure 3:**
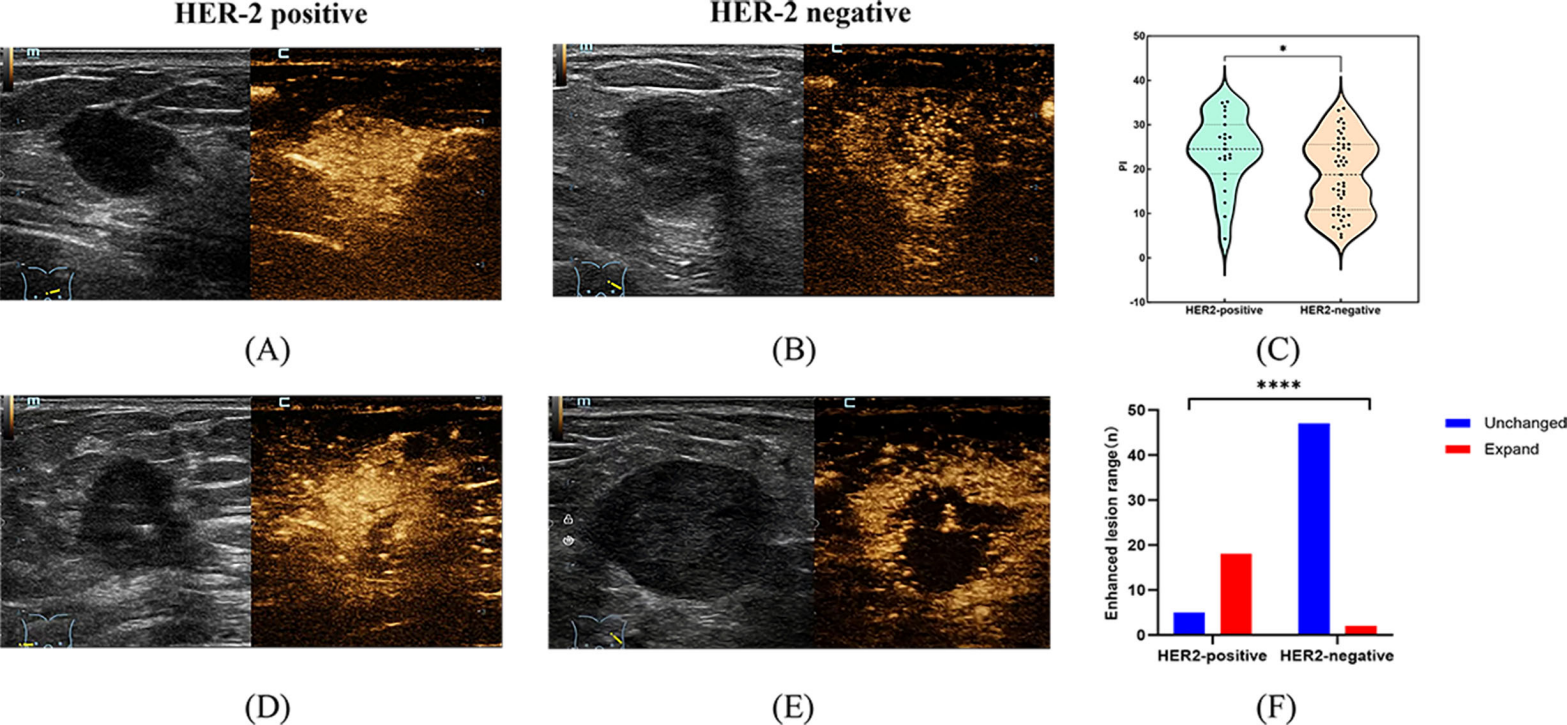
Representative images of peak intensity and area of enhancement in HER2-positive and HER2-negative breast masses. **(A, D)** show contrast-enhanced ultrasound images of HER2-positive breast masses; **(B, E)** show contrast-enhanced ultrasound images of HER2-negative breast masses. The violin plot **(C)** compares peak intensity between HER2-positive and HER2-negative groups. Corresponding bar graph **(F)** illustrates the quantitative comparison of area of enhancement between the two groups. * indicates *P*-value < 0.05, **** indicates *P*-value < 0.0001.

**Figure 4:**
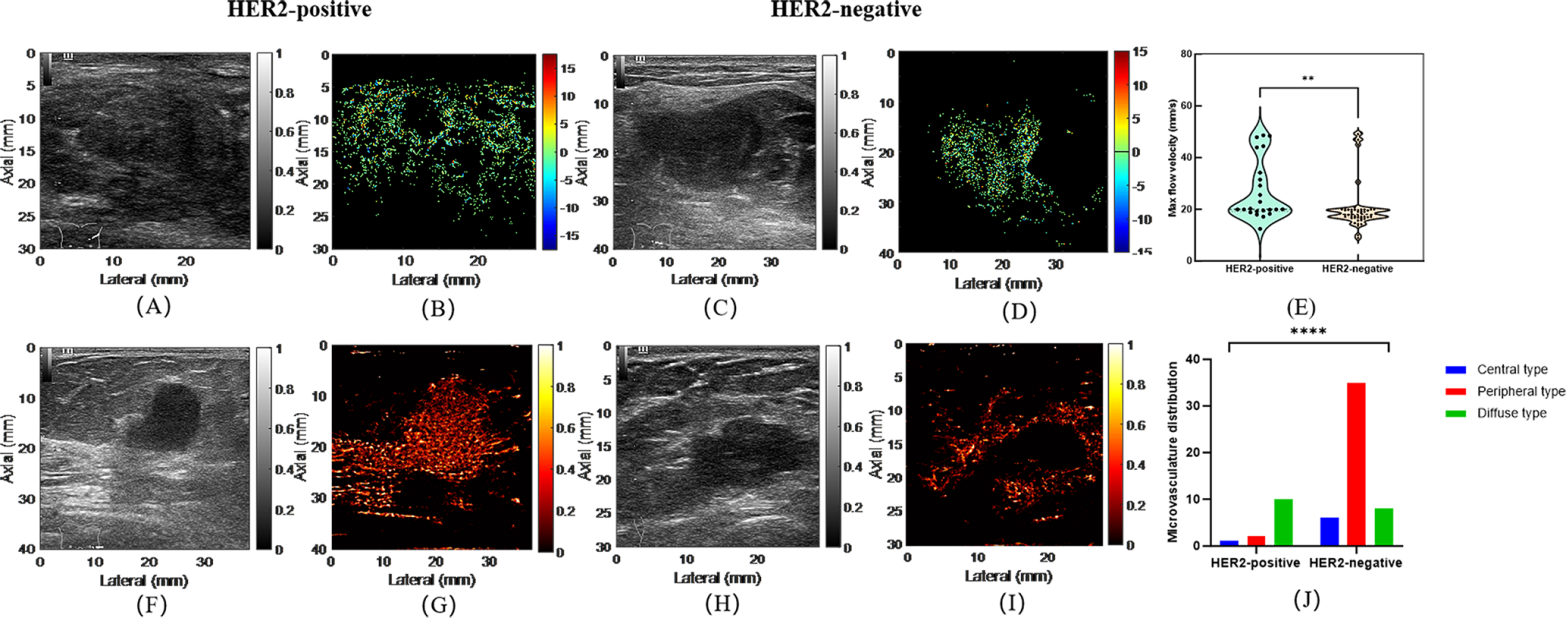
Representative images of microvasculature flow velocity and microvasculture distribution in HER2-positive and HER2-negative breast masses. **(A, B)** are conventional ultrasound image and microvasculature flow velocity image of a HER2-positive breast mass. **(C, D)** are conventional ultrasound image and microvasculature flow velocity image of a HER2-negative breast mass. The violin plot **(E)** illustrates the comparison of max flow velocity between HER2-positive and HER2-negative groups. **(F, G)** are conventional ultrasound image and microvasculature distribution image of a HER2-positive breast mass. **(H, I)** are conventional ultrasound image and microvasculature distribution image of a HER2-negative breast mass. The bar graph **(J)** illustrates the comparison of microvasculature distribution between HER2-positive and HER2-negative groups. **p < 0.01; ****p < 0.0001.

**Figure 5:**
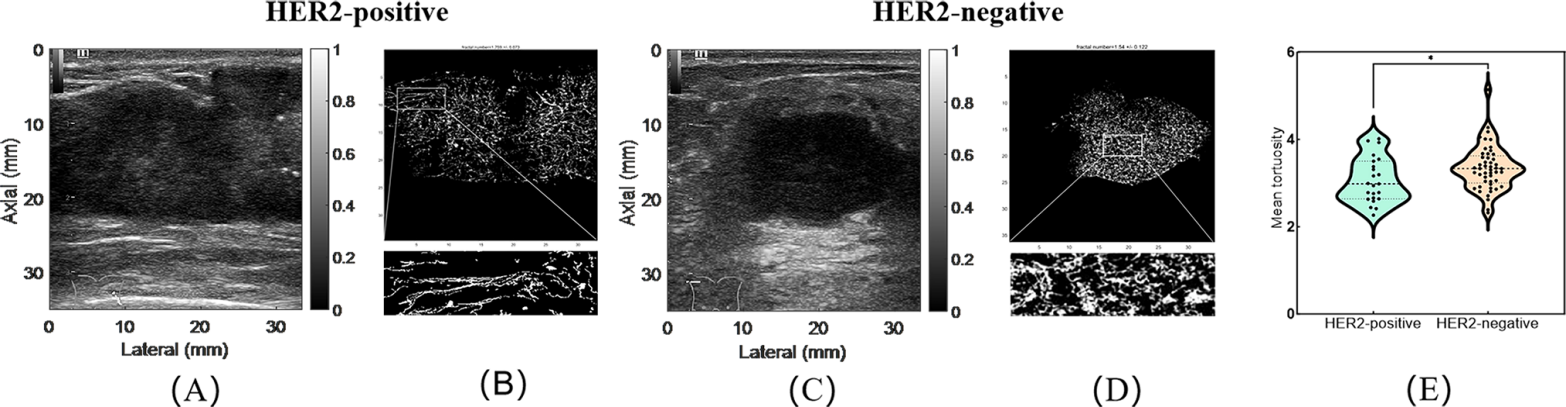
Representative images of microvasculature tortuosity in HER2 positive and HER2 negative breast masses. **(A, B)** are conventional ultrasound image and microvasculature tortuosity image of HER2-positive breast mass. **(C, D)** are conventional ultrasound image and microvasculature tortuosity image of HER2-negative breast mass. **(E)** is the corresponding violin plot. *p < 0.05.

Differences in qualitative and quantitative CEUS and SRUS parameters were evaluated between HER2-positive and HER2-negative breast cancers (**Tables 4**, **5**). The PI value was significantly higher in the HER2-positive group (29.47 ± 1.51 dB vs. 17.91 ± 1.14 dB, *p* < 0.001; **Figures 3A–C**). In terms of CEUS qualitative analysis, area of enhancement showed distinct differences as most HER2-positive group exhibited an enlarged enhancement area, whereas the majority of HER2-negative group demonstrated remained unchanged in size following enhancement (**Figures 3D–F**). Compared to the HER2-negative group, the HER2-positive group exhibited significantly higher max (20.0 cm/s [19.2-34.1 cm/s] vs. 18.0 cm/s [16.8-19.9 cm/s], *p* = 0.001) and mean flow velocity (6.0 cm/s [4.1-6.9 cm/s] vs. 4.1 cm/s [3.5-5.5 cm/s], *p* = 0.018) (**Figures 4A–E**). Regarding microvasculature distribution, peripheral distribution was predominant in both groups, but it was more frequently observed in HER2-negative breast cancer (**Figures 4F–J**). Conversely, vascular tortuosity was significantly lower in HER2-positive tumors, indicating straighter vessels (**Figure 5**).

**Table 4:** Comparison of CEUS parameters between HER2-positive and HER2-negative breast cancers.

Parameters	HER2-positive (n=23)	HER2-negative (n=49)	t/χ²/z	*P*
PI	24.4 (18.9,30.0)	18.7 (10.8,25.5)	-2.403	**0.016**
TTP	21.9 ± 4.3	20.9 ± 6.4	-0.819	0.416
AUC	906.8 (443.8,1256.0)	673.4 (331.2,1175.9)	-0.948	0.343
Wash-in rate	1.17 (0.9,1.4)	0.88 (0.71,1.23)	-2.392	**0.017**
Wash-out rate	-0.2 (-0.3,-0.2)	-0.2 (-0.3,-0.2)	-2.087	**0.037**
Wash-in rate/wash-out rate	-4.2 (-4.8,-3.6)	-4.5 (-5.1,-3.5)	-0.242	0.809
Enhancement time			0.964	0.618
Delayed enhancement	2 (8.7)	8 (16.3)		
Simultaneous enhancement	12 (52.2)	21 (42.9)		
Rapid enhancement	9 (39.1)	20 (40.8)		
Enhancement intensity			0.173	0.917
Hypoenhancement	1 (4.3)	2 (4.1)		
Isoenhancement	11 (47.8)	21 (42.9)		
Hyperenhancement	11 (47.8)	26 (53.1)		
Enhancement pattern			0.911	0.634
Centripetal enhancement	14 (60.9)	33 (67.3)		
Centrifugal enhancement	0 (0.0)	1 (2.0)		
Diffuse enhancement	9 (39.1)	15 (30.6)		
Distribution of contrast agent			0.115	0.735
Homogeneous distribution	6 (26.1)	11 (22.4)		
Heterogeneous distribution	17 (73.9)	38 (77.6)		
Enhancement margin			0.128	0.721
Clear	7 (30.4)	17 (34.7)		
Blurred	16 (69.6)	32 (65.3)		
Filling defect			0.026	0.872
No	8 (34.8)	18 (36.7)		
Yes	15 (65.2)	31 (63.3)		
Peripheral vessels penetrating the lesion			0.476	0.490
No	0 (0.0)	1 (2.0)		
Yes	23 (100.0)	48 (98.0)		
Area of enhancement			42.933	**<0.001**
Unchanged	5 (21.7)	47 (95.9)		
Larger	18 (78.3)	2 (4.1)		

Bold values indicate statistically significant differences (P < 0.05).

**Table 5:** Comparison of SRUS parameters between HER2-positive and HER2-negative breast cancers.

Parameters	HER2-positive (n=23)	HER2-negative (n=49)	χ²/z	*P*
Mean flow velocity	6.0 (4.1,6.9)	4.1 (3.5,5.5)	-2.373	**0.018**
Mean tortuosity	3.0 (2.6,3.5)	3.3 (3.0,3.6)	2.148	**0.035**
Mean diameter	35.3 (32.8,37.8)	35.6 (34.4,37.7)	0.647	0.520
Max flow velocity	20.0 (19.2,34.1)	18.0 (16.8,19.9)	-3.213	**0.001**
Max tortuosity	61.7 (33.8,89.4)	81.4 (49.5,121.2)	-1.890	0.059
Largest diameter	195.8 (147.5,251.7)	212.2 (171.0,243.9)	-0.628	0.530
Fractal dimension	1.4 (1.3,1.5)	1.4 (1.3,1.6)	-0.320	0.749
Microvasculature density	5.2 (2.9,8.3)	4.8 (2.5,7.0)	-0.779	0.436
Microvasculature distribution			6.509	**0.039**
Central type	1 (4.3)	6 (12.2)		
Peripheral type	12 (52.2)	35 (71.4)		
Diffuse type	10 (43.5)	8 (16.3)		
Microvasculature flow direction			13.064	**<0.001**
Toward the transducer	5 (21.7)	33 (67.3)		
Away from the transducer	18 (78.3)	16 (32.7)		

Bold values indicate statistically significant differences (P < 0.05).

### 3.4 Binary logistic regression analysis of CUS, CEUS, and SRUS parameters with HER2-positive and HER2-negative breast cancers

Binary logistic regression was conducted to evaluate the predictive value of ultrasound parameters for HER2-positive breast cancer. Maximum diameter of the lump, microcalcification, PI, area of enhancement, mean tortuosity, maximum flow velocity, microvasculature distribution, and microvasculature direction were eight risk factors for ultrasound prediction of HER2-positive breast cancer, with microvasculature flow direction exhibiting the highest odds ratio (**Table 6**).

**Table 6:** Binary logistic regression results of ultrasound parameters for differentiating HER2-positive and HER2- negative breast cancers.

Parameters	β	S.E	Wald	*P*	OR	95% C.I. for OR	
						Lower	Upper
Maximum diameter of the lump (cm)	0.378	0.190	3.987	0.046	1.460	1.007	2.117
Microcalcification	1.167	0.551	4.491	0.034	3.212	1.092	9.450
PI	0.079	0.033	5.557	0.018	1.082	1.013	1.155
Area of enhancement	-4.438	0.881	25.353	<0.001	0.012	0.002	0.067
Mean tortuosity	-1.170	0.569	4.224	0.040	0.311	0.102	0.947
Max flow velocity	0.052	0.024	4.754	0.029	1.053	1.005	1.103
Microvasculature distribution	1.167	0.495	5.566	0.018	3.211	1.218	8.465
Microvasculature flow direction	2.005	0.590	11.538	<0.001	7.425	2.335	23.610
Constant	-5.694	5.185	1.206	0.274	0.003		

### 3.5 Diagnostic performance of CUS, CEUS, and SRUS alone and in combination

**Figure 6:**
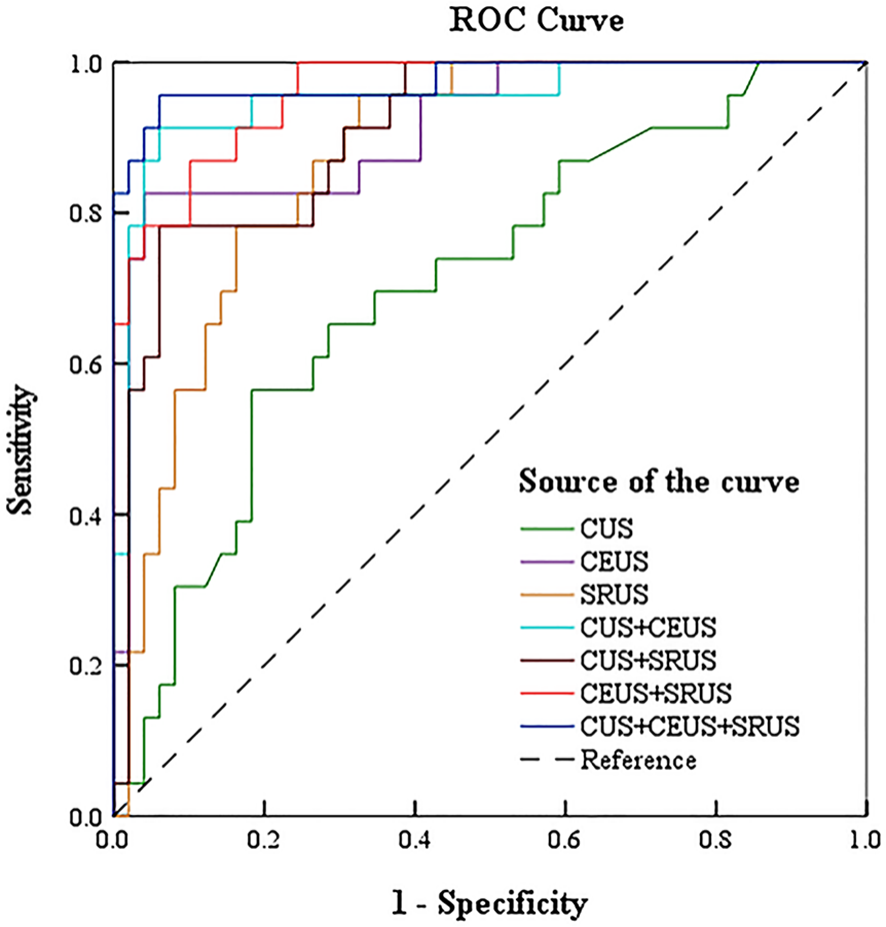
The receiver operating characteristic curves for diagnosis of HER2-positive and HER2-negative breast cancers by conventional ultrasound, enhanced ultrasound, super-resolution ultrasound imaging alone and in combination.

ROC analysis evaluated the diagnostic performance of seven imaging approaches in distinguishing HER2-positive from HER2-negative breast cancers, including CUS, CEUS, SRUS, CUS+CEUS, CUS+SRUS, CEUS+SRUS, and CUS+CEUS+SRUS. Among the individual modalities, CEUS showed the highest specificity (95.9%), accuracy (90.3%), and AUC (0.914), whereas SRUS achieved the highest sensitivity (95.7%) but a comparatively lower AUC of 0.874. The three-modality combination (CUS + CEUS + SRUS) yielded an AUC of 0.976, with a sensitivity of 95.7% and specificity of 93.9%, outperforming dual-modality combinations (**Table 7**). **Figure 6** shows the ROC curves.

**Table 7:** The diagnostic efficacy of CUS, CEUS, SRUS and their combination in differentiating HER2-negative and HER2-positive breast cancers.

Group	Sensitivity (%)	Specificity (%)	Accuracy (%)	AUC	95% C.I.
CUS	59.5	81.6	72.2	0.705	0.577-0.834
CEUS	82.6	95.9	90.3	0.914	0.841-0.987
SRUS	95.7	67.3	79.2	0.874	0.795-0.953
CUS+CEUS	91.3	93.9	93.1	0.951	0.894-1.000
CUS+SRUS	78.5	93.9	87.5	0.907	0.837-0.977
CEUS+SRUS	87.0	89.8	88.9	0.960	0.921-0.999
CUS+CEUS+SRUS	95.7	93.9	94.4	0.976	0.938-1.000

## 4 Discussion

In this study, we extracted feature parameters from multiple ultrasound modalities and analyzed their differences and diagnostic performance in distinguishing HER2-positive from HER2-negative breast cancers. Binary logistic regression analysis revealed that maximum diameter of the lump, microcalcification, PI, area of enhancement, max flow velocity, mean tortuosity, microvasculature distribution, and microvasculature flow direction were significantly associated with HER2 status, with microvasculature flow direction showing the strongest association. In addition, the ROC analysis demonstrated that the combination of CUS, CEUS, and SRUS achieved the highest AUC compared to individual or dual-modality approaches, providing valuable information for early identification of HER2-positive breast cancer and aiding clinical decision-making.

Compared with HER2-negative breast cancer, HER2-positive breast cancer has higher malignancy, more aggressive tumor growth, increased risk of metastasis and recurrence, and worse prognosis (27). Currently HER2-targeted therapies, including monoclonal antibodies and tyrosine kinase inhibitors, have been shown to significantly improve patient outcomes (28, 29). CUS is a primary imaging modality for breast cancer, and previous studies have demonstrated its potential in predicting the molecular subtype and HER2 status (30, 31). In this study, HER2-positive tumors were larger, with microcalcifications and parallel growth, consistent with prior findings. Previous studies have shown that mass size and microcalcification were key predictors of HER2 status (32, 33). HER2 promotes tumor cell proliferation, causing local ischemia that induces cell death and microcalcification. Related studies have shown that HER2-positive tumors alter immune cell activity, increasing BMP-2 levels and contributing to calcification (34, 35). Consequently, HER2-positive breast cancer typically exhibits a larger size, with microcalcifications and parallel growth.

Breast cancer is an angiogenesis-dependent tumor with high heterogeneity, and its molecular subtypes exhibit distinct vascular patterns. Related studies have shown that HER2 overexpression promotes angiogenesis by upregulating VEGF and stimulating endothelial cell proliferation, resulting in higher MVD in HER2-positive tumors (36, 37). In this study, CDFI showed no significant difference in adler grade between HER2-positive and HER2-negative groups, differing from Zhu et al. and Wang et al., who reported richer blood flow in HER2-positive tumors (37, 38). The discrepancy may be attributed to differences in grouping strategies. Zhu et al. classified breast cancer into four molecular subtypes, Wang et al. divided adler grade into two groups, while this study divided breast cancer into HER2-positive and HER2-negative groups, and divided adler grade into four groups. Differences in grouping strategies may partially account for the inconsistencies in findings. Further multicenter studies with larger cohorts are needed to clarify the relationship between CDFI features and HER2 status.

CEUS is widely used and safe diagnostic tool in breast cancer, allowing continuous visualization of blood flow and microcirculation, thereby improving lesion detection and diagnostic accuracy (18, 39). It has also been applied to assess HER2 expression (30, 40). In this study, PI derived from the TIC analysis on CEUS was significantly higher in HER2-positive tumors, which also exhibited faster wash-in and wash-out perfusion and greater lesion extent following contrast enhancement. The results of this study are consistent with the results of previous studies. The study of Li et al. showed that HER2-positive breast cancer had a larger PI (40). Wang et al. proved that HER2-positive breast cancers had greater AS and the overranging phenomenon (41). Li et al. demonstrated that the HER2 overexpression subtype showed rapid enhancement (95.65%) and larger lesion size after enhancement (100%), and higher PI compared to the Luminal A and Luminal B subtypes (42). Park et al. performed low-dose perfusion computed tomography (CT) in 70 breast cancer patients and found that HER2-overexpressing breast cancers had significantly higher perfusion and PI (43). This phenomenon may be related to VEGF-mediated angiogenic patterns reported in previous studies (44–47), although direct molecular validation was not performed in the present study. Such vascular characteristics could contribute to faster perfusion and changes in tumor blood flow. In addition, PI rises with increasing tumor neovascularization, and high-perfusion breast cancers tend to show greater lesion extent after CEUS (47, 48).

SRUS overcomes the ultrasound diffraction limit to visualize tumor microvasculature, enabling quantitative and qualitative assessment of morphological and functional parameters of microcirculation (49). Our previous studies have shown that SRUS can provide valuable microvasculature information for distinguishing between benign and malignant breast masses (25, 50), but its role in predicting molecular subtypes remains unexplored. Zheng et al. proposed a protocol to evaluate the relationship between SRUS parameters and histologic biomarkers in breast cancer, but the study is still in progress (51). This study demonstrated faster microvasculature flow velocity in HER2-positive breast cancer. And qualitative analysis showed that HER2-positive breast cancers had more uniform microvasculature distribution, smaller tortuosity, and flow directed away from the transducer. These findings are supported by Park et al., who reported increased perfusion function and blood volume permeability in HER2-positive tumors using low-dose perfusion CT, suggesting faster flow and uniform distribution (52). Kim et al. evaluated the correlation between tumor vascularity and prognostic biomarkers in breast cancer patients using CT and MRI. The results showed that HER2 expression was significantly associated with blood flow perfusion, indicating increased angiogenesis and higher flow rates (53). A study using incoherent motion diffusion-weighted imaging showed that HER2-positive breast cancer had higher blood flow velocity and capillary number (54). Mean vessel tortuosity is a method to quantify uncontrolled vasculature, measuring the abnormal twisting of vessels and indicating the complexity of vascular pathways (55). Previous studies have shown that tumor neovascular tortuosity can block blood supply (56). This may further affect blood flow velocity. The result of this study suggested that HER2-positive breast cancers exhibited faster blood flow, suggesting lower intratumoral tortuosity. Previous studies have shown that CDFI can detect blood flow direction (57–59), but it cannot resolve microvessels with small diameter or low velocity. In this study, we used SRUS to track the direction of microbubble movement and found that microvasculature flow in HER2-positive breast cancer was away from the transducer. These results provided more information on microcirculation for noninvasive prediction of HER2-positive breast cancer, and provided a basis for clinicians to make treatment decisions in advance.

HER2 expression has been reported to positively correlate with VEGF expression and tumor angiogenesis, thereby promoting neovascular formation and increased vascular permeability in breast cancer (34, 43, 60, 61). In this study, we evaluated the value of multimodal ultrasound in predicting HER2-positive breast cancer. As a pure blood pool imaging technique, CEUS enables real-time monitoring of the entire tissue perfusion process (62, 63). SRUS overcomes the ultrasound diffraction limit to display the morphology and structure of microvessels in tumors (64). The results of the study showed that the combination of CUS, CEUS, and SRUS achieved the highest diagnostic performance, outperforming individual or dual-modality methods. The combination of CEUS and SRUS enabled visualization of millimeter and micron level tumor vessels, integrating CUS features and enhancing diagnostic efficacy for predicting HER2-positive breast cancer, thereby providing a robust basis for clinical decision-making.

However, this study has several limitations. First, it focused solely on differences in multimodal ultrasound parameters between HER2-positive and HER2-negative breast cancers. Future research could incorporate four molecular subtypes to further elucidate the imaging characteristics specific to different molecular subtypes. Second, the sample size of this study is small, and the findings require validation in larger cohorts. Third, HER2 status was determined by immunohistochemistry using the EnVision™ system, with equivocal cases confirmed by FISH. However, variability in IHC sensitivity related to differences in antibody clones, detection systems, and staining protocols may influence classification, particularly in cases with low HER2 expression. Fourth, the blood flow parameters extracted by vascular imaging are relatively limited. In future studies, more high-throughput parameters can be extracted from dynamic CEUS video and 3D ultrasound SRUS through radiomics.

## 5 Conclusion

In conclusion, the multimodal ultrasound model (CUS, CEUS, and SRUS) has shown good predictive efficacy in distinguishing HER2-positive and HER2-negative breast cancer. As a non-invasive method, it provides a scientific basis for treatment planning and is crucial for improving patient prognosis.

## Data Availability

The raw data supporting the conclusions of this article will be made available by the authors, without undue reservation.
